# Alzheimer’s disease polygenic risk score associates with hippocampal subfield atrophy and immune-related genetic mechanism

**DOI:** 10.3389/fnagi.2026.1875172

**Published:** 2026-08-05

**Authors:** Clàudia Olivé, Itziar de Rojas, Linda Zhang, Oscar Sotolongo-Grau, Inés Quintela, Pablo García-González, Raquel Puerta, Laura Montrreal, Paula Bayón, Marta Rovira, Alejandro Valenzuela, Maria Capdevila-Bayo, Álvaro Muñoz, Andrea Miguel, Berta Calm, Miguel Calero, Alberto Rábano, Ana Belén Pastor, Teodoro del Ser, Miguel Medina, Ángel Carracedo, Alfredo Ramírez, Laura Molina-Porcel, Xavier Montalbán, Amanda Cano, Sergi Valero, Marta Marquié, Pilar Sanz, Pascual Sánchez-Juan, Mercè Boada, Bryan Strange, Maria Victoria Fernández, Agustín Ruiz

**Affiliations:** 1 Ace Alzheimer Center Barcelona - Universitat Internacional de Catalunya, Barcelona, Spain; 2 Facultat de Farmàcia i Ciències de l’Alimentació, Universitat de Barcelona, Barcelona, Spain; 3 Luxembourg Centre for Systems Biomedicine, University of Luxembourg, Esch-sur-Alzette, Luxembourg; 4 CIBERNED, Network Center for Biomedical Research in Neurodegenerative Diseases, National Institute of Health Carlos III, Madrid, Spain; 5 Alzheimer’s Centre Reina Sofia-CIEN Foundation-ISCIII, Madrid, Spain; 6 Grupo de Medicina Xenómica, Centro Nacional de Genotipado (CEGEN-PRB3-ISCIII), Universidad de Santiago de Compostela, Santiago de Compostela, Spain; 7 Fundación Pública Galega de Medicina Xenómica- CIBERER-IDIS, Santiago de Compostela, Spain; 8 Division of Neurogenetics and Molecular Psychiatry, Department of Psychiatry and Psychotherapy, Faculty of Medicine and University Hospital Cologne, University of Cologne, Cologne, Germany; 9 Department of Neurodegenerative Disease and Geriatric Psychiatry, University of Bonn Medical Center, Bonn, Germany; 10 German Center for Neurodegenerative Diseases (DZNE), 3A Von-Siebold-Str, Göttingen, Germany; 11 Excellence Cluster on Cellular Stress Responses in Aging-Associated Diseases (CECAD), University of Cologne, Cologne, Germany; 12 Glenn Biggs Institute for Alzheimer’s and Neurodegenerative Diseases, University of Texas Health Sciences Center, San Antonio, TX, United States; 13 Alzheimer’s Disease and Other Cognitive Disorders Unit, Neurology Service, Hospital Clínic, I Fundació de Recerca Clínic Barcelona-Institut d’Investigacions Biomèdiques August Pi i Sunyer (FRCB-IDIBAPS) and Institute of Neurosciences, University of Barcelona, Barcelona, Spain; 14 Neurological Tissue Bank, Biobanc-Hospital Clínic-FRCB/IDIBAPS, Barcelona, Spain; 15 Multiple Sclerosis Centre of Catalonia and Department of Neurology, Hospital Universitari Vall d'Hebron, Universitat Autònoma de Barcelona, Barcelona, Spain; 16 Laboratory for Clinical Neuroscience, Centre for Biomedical Technology, Universidad Politécnica de Madrid, IdISSC, C. del Prof Martín Lagos, Madrid, Spain; 17 Department of Microbiology, Immunology and Molecular Genetics, Long School of Medicine, University of Texas Health Science Center, San Antonio, TX, United States

**Keywords:** Alzheimer’s disease, hippocampal sclerosis of aging, hippocampal subfield volumes, magnetic resonance imaging, polygenic risk score

## Abstract

**Introduction:**

Hippocampal atrophy is frequently observed in neurodegenerative diseases such as Alzheimer’s disease (AD) or hippocampal sclerosis of aging (HS-aging). Volume loss in the hippocampus is described as prodromal stage of dementia and has been associated with AD polygenic risk score (PRS). CA1 and subiculum atrophy have been suggested to be a promising *in vivo* biomarker for HS-aging. Recent studies suggest that some loci associated with AD may be more related to other brain diseases concomitant with AD. We aimed to find which significant single nucleotide polymorphisms (SNPs) in the latest AD genome wide association studies (GWAS) could be potentially related with early atrophy of specific hippocampal subregions related to HS-aging.

**Materials and methods:**

We used regression models to assess the relation of the AD-PRS, and genome-wide significant AD variants, with CA1 and subiculum volumes assessed by magnetic resonance imaging (MRI) in 1,859 participants without dementia (with mild cognitive impairment or cognitively healthy). Co-regulatory network analyses and over-representation enrichment analyses were conducted to identify biological pathways enriched with co-regulatory networks of genes associated with hippocampal subregion volumes. We meta-analyzed data from seven cohorts to associate their AD-PRS with AD in the presence of concomitant brain pathologies.

**Results:**

Reduced volumes of CA1 and subiculum show association with higher levels of AD-PRS and with variant rs5848 in *GRN*. This variant was enriched in immune-related pathways. AD-PRS showed no significant association with AD pathology alone, but was strongly associated with AD in the presence of concomitant neurodegenerative pathologies, with HS-aging showing the largest effect size.

**Discussion:**

Specific AD-SNPs enriched in immune-brain axis pathways rather than A*β* related processes, were associated with reduced volumes of CA1 and subiculum prior to dementia onset. This supports that AD-PRS may capture genetic susceptibility to concomitant neurodegenerative diseases frequently misdiagnosed as AD. Given that AD-PRS is most strongly associated with AD in cases with HS-aging, and that CA1 and subiculum are promising *in vivo* biomarker for HS-aging also linked with rs5848 in *GRN*, our findings are consistent with HS-aging related vulnerability. This insight links early hippocampal subfields atrophy to shared genetic mechanisms and biological pathways of interest.

## Introduction

Alzheimer’s disease (AD) is the most common cause of dementia and a highly heterogeneous neurodegenerative disorder, with variation in symptom onset, severity, and progression. Its clinical presentation is often complex due to the presence of multiple underlying neuropathologic comorbidities. Consequently, genome-wide association studies (GWAS) based on clinically diagnosed AD have provided limited capacity to translate genetic findings into potential targeted therapies, as several loci identified in prior studies are also associated with other AD-related dementias (Shade et al., 2024).

Over the past decade, GWAS have expanded markedly in both sample size and the breadth of traits examined. Large international collaborative efforts (Lambert et al., 2013; Kunkle et al., 2019; de Rojas et al., 2021; Bellenguez et al., 2022), have substantially advanced our understanding of AD genetics, raising the number of known risk loci to more than 80 (Bellenguez et al., 2022). However, adding a larger sample size of clinically labeled individuals may result in the addition of cases with co-pathologies, and often misdiagnosis (Pao et al., 2011; de Rojas et al., 2021; Escott-Price and Hardy, 2022). Indeed, over half of patients diagnosed with AD harbor additional brain pathologies contributing to cognitive impairment, including hippocampal sclerosis of aging (HS-aging), vascular lesions, Lewy bodies (LBD), TDP-43 inclusions, or argyrophilic grains (Schneider et al., 2007). Therefore, focusing on neuropathological endophenotypes may reduce diagnostic heterogeneity and provide a clearer view of the distinct genetic architectures underlying each co-pathology, ultimately refining our interpretation of AD risk loci.

Although hippocampal atrophy is a widely used and validated biomarker of AD (de Flores et al., 2015), it lacks disease specificity, as volume loss is also observed in frontotemporal dementia (FTD), limbic-predominant age-related TDP-43 encephalopathy (LATE), LBD and HS-aging (Kantarci et al., 2016; Pini et al., 2016; Nelson et al., 2019; Woodworth et al., 2021; Ortega-Cruz et al., 2023; Planche et al., 2023; Kim et al., 2025). However, the hippocampus is composed of anatomically and functionally distinct subfields that differ in their contributions to memory processing and their vulnerability to neurodegeneration. Consequently, studying individual hippocampal subfields may improve the specificity of MRI measures of atrophy as biomarkers of distinct neuropathologies. Furthermore, reductions in hippocampal volume are detectable in individuals with mild cognitive impairment (MCI), a clinically heterogeneous syndrome that frequently represents a prodromal stage of dementia. Progressive reductions in hippocampal volume, particularly within specific hippocampal subfields, have been associated with cognitive decline and an increased risk of conversion from MCI to dementia (Convit et al., 1997; Hampel et al., 2014; Kantarci et al., 2016; Zanchi et al., 2017). These findings indicate that hippocampal neurodegeneration begins well before the onset of overt dementia. This likely reflects the convergence of multiple pathological processes on a vulnerable medial temporal lobe network. Consequently, identical neuroimaging phenotypes may arise from distinct underlying pathologies. Disentangling the molecular and genetic drivers contributing to hippocampal atrophy across different disease contexts is therefore essential to refine its biological interpretation and improve its utility for early risk stratification.

HS-aging is present in a considerable proportion of individuals over 85 years old who die with dementia (Nelson et al., 2013). The main pathological features of HS-aging include severe neuronal loss and gliosis in specific regions of the hippocampus, leading to cognitive and memory symptoms that appear to mimic AD. Recent evidence suggests that atrophy in the hippocampal subregions, CA1 and subiculum, measured by MRI, represents a promising *in vivo* biomarker for HS-aging (Woodworth et al., 2021; Ortega-Cruz et al., 2023), with hippocampal atrophy appearing earlier in HS-aging than in AD (Li et al., 2021). Although its etiology remains largely unknown, various studies support a genetic contribution to HS-aging risk (Dickson et al., 2010; Tsai et al., 2013; Katsumata et al., 2017; Dugan et al., 2021; Shade et al., 2024). Notably, AD polygenic risk scores (AD-PRS) have been reported to be more strongly associated with HS-aging co-occurring with AD than with AD alone (de Rojas et al., 2021), and are also associated with reduced hippocampal subfield volumes in cognitively healthy individuals (Harrison et al., 2016; Foo et al., 2021). Collectively, these observations underscore the sensitivity of hippocampal atrophy to early neurodegenerative processes and raise the possibility that shared genetic mechanisms may contribute to hippocampal vulnerability across distinct pathologies.

In this study, we hypothesized that specific variants included in the AD-PRS (Bellenguez et al., 2022) are associated with MRI-measured volumes of CA1 and subiculum, proxies for HS-aging, prior to dementia onset. Therefore, for the analyses presented here, we used hippocampal subfield volumes CA1 and subiculum (Woodworth et al., 2021; Ortega-Cruz et al., 2023) measured with MRI, in participants without a current dementia diagnosis (MCI and cognitively healthy individuals), and assessed their association with variants included in the latest AD-PRS (Bellenguez et al., 2022). We designed our analyses to detect which AD-related SNP might be more specifically associated with prodromal hippocampal subfield atrophy related to HS-aging. We examined both their individual and combined effects, and investigated their relation with biological pathways and brain pathologies concomitant with AD.

## Materials and methods

### Participants

For this study we focused on individuals with mild cognitive impairment (MCI), who are at increased risk of developing dementia, and cognitively healthy individuals. By focusing on this early stage, we aim to identify genetic variants associated with lower hippocampal volumes before the onset of dementia symptoms. We leveraged data from Ace Alzheimer Center Barcelona (ACE) (Boada et al., 2014), Alzheimer’s Disease Neuroimaging Initiative [ADNI, adni.loni.usc.edu/, Mueller et al. (2005); Beckett et al. (2015)] and the Vallecas Project (VP) (Olazarán et al., 2015). All samples for this study have available MRI, array genetic data and clinical information.

A total of 263 participants included in the study are from ACE. Specifically 56 MCI individuals (66.07% females and average age of 61.46 (±3.48) years) were collected through the BIOFACE project (Esteban De Antonio et al., 2021), 2 MCI individuals and 156 controls (62.66% females and average age of 67.27 (±6.90) years) from Fundació ACE Healthy Brain Initiative (FACEHBI) (Rodriguez-Gomez et al., 2017), 23 MCI individuals and 6 controls (51.72% females and average age of 70.68 (±5.82) years) from the European Prevention of Alzheimer’s Dementia (EPAD) (Ritchie et al., 2020) and 19 MCI individuals and 1 control (45.00% females and average age of 73.79 (±4.63) years) from Models of Patient Engagement for Alzheimer’s Disease (MOPEAD) (Rodríguez-Gómez et al., 2019). From ADNI we selected 353 MCI individuals and 209 controls (39.32% females and average age of 75.12 (±6.51) years) from ADNI1 and 212 MCI individuals and 93 controls (46.23% females and average age of 71.89 (±7.24) years) from ADNI2. Finally, we also leveraged a cohort of 729 controls (68.04% females and average age of 74.74 (±3.84) years) from the VP (Olazarán et al., 2015). A summary of demographic and clinical information for the participants included in this study by project is shown in Table 1, and further information about each individual project is provided in the Supplementary material. All projects passed the ethics committee and written informed consent was obtained from all participants. All protocols were approved by the Clinical Research Ethics Commission of the Hospital Clinic, Barcelona, Spain (reference: HCB/2014/0494) in accordance with the current Spanish regulations in the field of biomedical research and the Declaration of Helsinki.

**Table 1:** Demographic and clinical data for the cohorts included in this study by project.

Cohorts	*N*	Females (%)	MCI (%)	CO (%)	Age (±SD)	Education (±SD)
ADNI-1	562	221(39.32)	353 (62.81)	209 (37.19)	75.12 (6.51)	15.83 (2.97)
ADNI-2	305	141 (46.23)	212 (69.51)	93 (30.49)	71.89 (7.24)	16.16 (2.58)
BIOFACE	56	37 (66.07)	56 (100)	00 (00.00)	61.46 (3.48)	9.77 (3.05)
FACEHBI	158	99 (62.66)	2 (1.27)	156 (98.73)	67.27 (6.90)	12.98 (4.59)
EPAD	29	15 (51.72)	23 (79.31)	6 (20.69)	70.68 (5.82)	9.79 (3.48)
MOPEAD	20	9 (45.00)	19 (95.00)	1 (5.00)	73.79 (4.63)	9.10 (4.06)
VP	729	496 (68.04)	00 (00.00)	729 (100)	74.74 (3.84)	10.30 (5.77)
Total	1,859	1,018 (54.76)	665 (35.77)	1,194 (64.22)	73.28 (6.47)	13.13 (5.17)

* N, number of samples; MCI, mild cognitive impairment; CO, Controls; Age, average age at MRI; SD, standard deviation.

Finally, 332 patients with neuropathological confirmation of AD and genetic data available (from the European Alzheimer’s and Dementia Biobank, ACE and Banc de Teixits Neurologics (BTN) at Fundació de Recerca Clínic Barcelona-Institut d’Investigacions Biomèdiques August Pi i Sunyer (FRCB-IDIBAPS; Supplementary material), was used to re-evaluate and extend the association between AD-PRS and AD concomitant pathologies previously published by de Rojas et al. (2021)) using an updated AD-PRS (Bellenguez et al., 2022). Details are provided elsewhere (de Rojas et al., 2021).

### Genomic data processing, QC and AD-PRS

Samples from ACE and VP were genotyped as part of the Genome Research at Ace Alzheimer center (GR@ACE) and Dementia Genetics Spanish Consortium (DEGESCO) genetic initiatives (Moreno-Grau et al., 2019; de Rojas et al., 2021). Genotyping was conducted using the Axiom 815 K Spanish biobank array (according to manufacturer’s instructions - Axiom™ 2.0 Assay Manual Workflow, Thermo Fisher) at the Spanish National Center for Genotyping (CeGEN, Santiago de Compostela, Spain). Details on genotyping and quality control procedures are provided elsewhere (Moreno-Grau et al., 2019).

ADNI samples were genotyped with the Illumina Human 610-Quad BeadChip (Illumina, Inc., San Diego, CA, USA), for ADNI-1 and with the OmniExpress BeadChip for ADNI-GO/2 individuals (ADNI-2) (Saykin et al., 2010, 2015).

Genomic data quality control (QC) was performed uniformly in three parallel batches for ADNI-1, ADNI-2 and ACE-VP. QC included removal of samples with low-quality genotyping, excess of heterozygosity or high missingness and variants with call rate below 95% or deviation from the Hardy–Weinberg equilibrium (*p* value < 1 × 10^−06^). Additionally, samples with discordant genetic sex annotation, family members (PI-HAT > 0.1875), or with a non-European ancestry (as per 1,000 Genomes Project) were excluded from the analysis.

To maximize the AD-PRS coverage, imputed data was generated with the Trans-Omics for Precision Medicine (TOPMed) reference panel (Das et al., 2016; Taliun et al., 2021) on genome build GRCh38. Rare variants (minor allele frequency; MAF < 1%) and variants with low imputation quality (R^2^ < 0.3) were excluded.

A weighted individual AD-PRS was calculated based on the 83 genome-wide significant (GWS, *p* value < 5 × 10^−08^) variants reported by the European Alzheimer’s and Dementia Biobank (EADB) (Bellenguez et al., 2022). AD-PRS was generated by multiplying the genotype dosage of each risk allele for each variant by its respective weight and then summing across all variants. Due to its large effect, we excluded *APOE* variants (rs429358 and rs7412) from the AD-PRS calculation as well as the *ABI3* locus (rs616338) because it was not properly imputed in the ADNI-2 dataset.

### Structural MRI image acquisition

The image acquisition process was slightly different for each project of the ACE cohort included in this study. MRI scans from FACEHBI were performed on a 1.5 T Siemens MAGNETOM Aera (Erlangen, Germany), while for BIOFACE, EPAD and MOPEAD images were acquired with a Siemens MAGNETOM VIDA 3 T scanner (Erlangen, Germany) using a 32-channel head coil at Clínica Corachan, Barcelona. Anatomical T1-weighted images were acquired using a three-dimensional (3D) magnetization-prepared rapid gradient-echo (MPRAGE) sequence with different parameters for FACEHBI (repetition time (TR) 2.200 ms, echo time (TE) 2.66 ms, inversion time (TI) 900 ms, flip angle 8°, field of view (FOV) 250 mm, slice thickness 1 mm, and isotropic voxel size 1 × 1 × 1 mm), BIOFACE (TR 2.200 ms, TE 2.23 ms, TI 968 ms, 1.2 mm slice thickness, FOV 270 mm, and voxel measurement 1.1 × 1.1 × 1.2 mm), MOPEAD (TR 2.200 ms, TE 2.33 ms, TI 968 ms, flip angle 8°, FOV 270 mm, slice thickness 1.2 mm, and isotropic voxel size 1.1 × 1.1 × 1.2 mm) and EPAD (TR 2.300 ms, TE 2.93 ms, TI 900 ms, flip angle 9°, FOV 270 mm, slice thickness 1.2 mm, and isotropic voxel size 1.1 × 1.1 × 1.2 mm). Axial T2-weighted, 3D isotropic fast fluid-attenuated inversion recovery (FLAIR) and axial T2*-weighted sequences were also acquired to detect significant vascular brain damage or microbleeds.

All the subjects with existing GWAS information were chosen from ADNI cohorts (http://adni.loni.usc.edu/). The closest MRI to the baseline was selected for each subject. All MRI T1-weighted images were downloaded in NIfTI format (Jack et al., 2010, 2015). Since the ADNI protocol for MRI included one non-accelerated and one accelerated version of the scan, if available, more than one T1-weighted MRI for the same experiment was downloaded, in order to perform movement correction in the Freesurfer image processing pipeline (described below).

For the VP cohort, all T1-weighted images (3D fast spoiled gradient echo with inversion recovery preparation) were acquired using a 3 T MRI (Signa HDxt GEHC, Waukesha, USA) with a phased array 8 channel head coil and the following parameters: TR 10 ms, TE 4.5 ms, TI 600 ms, FOV 240 mm, matrix 288×288 and slice thickness 1 mm, yielding 0.5×0.5×1 mm voxel size. All MRI scans were reported by a neuroradiologist.

### Hippocampal subfields extraction

In order to extract the hippocampal subfield volumes, subjects from ACE (FACEHBI, BIOFACE, EPAD and MOPEAD) and ADNI (ADNI1 and ADNI2) were processed the same way. First, cortical reconstruction and volumetric segmentation for MRI images was performed with the Freesurfer 7.2 image analysis suite, which is documented and freely available for download online (https://surfer.nmr.mgh.harvard.edu/). The technical details of these procedures are described in prior publications (Dale et al., 1993, 1999; Sled et al., 1998; Fischl and Dale, 2000; Fischl et al., 2001, 2002, 2004; Ségonne et al., 2004, 2007; Reuter et al., 2010). Freesurfer segmentation was performed using both T1 and T2-weighted images for the ACE samples, while only T1-weighted images were used for the ADNI cohort.

Then, the hippocampal subfields segmentation was carried out using the hippocampal parcellation method included in Freesurfer 7.2 (Iglesias et al., 2015). Individual hippocampal subfield volumes for cornu ammonis (CA) were extracted out from results and grouped into a table. The following subfields of the hippocampal formation were used for the analyses in this study: CA1 body and head, subiculum body and head (Woodworth et al., 2021; Ortega-Cruz et al., 2023). Also, estimated total intracranial volumes (TIV) were extracted, from prior Freesurfer analyses, in order to make further volumetric corrections.

For the VP cohort, automatic segmentation of the hippocampus was performed on each participant’s T1-weighted image using FreeSurfer v.6.0 (https://surfer.nmr.mgh.harvard.edu/). Technical details of the whole-brain segmentation methods have been described previously (Fischl et al., 2002). Hippocampal volumes were extracted using the hippocampal subfields module in FreeSurfer 6.0 (Iglesias et al., 2015), and segmentations for all participants were visually inspected for accuracy.

### Association of AD-PRS with HS-aging related MRI

To account for MRI heterogeneity, hippocampal subfield volumes were standardized within each of the seven cohorts included in this study using the scale function in R (Supplementary Figure 1). Additionally, outliers differing ±3 SD from the mean were removed. In total, 17 samples were removed (10 subiculum outliers, 5 CA1 outliers, and 2 affecting both regions). Analyses were conducted using the volumes of CA1 and subiculum as an HS-aging related MRI phenotype (Woodworth et al., 2021; Ortega-Cruz et al., 2023) which we will be referring to as HS-MRI in this study. AD-PRS data was also standardized and independent t-tests were conducted to assess differences between AD-PRS means in individuals classified by diagnosis and *APOE* ɛ4 carriers. R version 4.1.2 software was used for all analyses.

Association of AD-PRS with HS-MRI was assessed using linear regression for each cohort. HS-by-proxy and AD-PRS were set as dependent and independent variables respectively, with sex, age, age^2^ (to account for the non-linear effect of age), years of education, diagnosis (when applicable), TIV and first ten genetic principal components (PC1-10; to adjust genetic variability for population structure) as covariates in the following regression model:


$$\begin{array}{l} \mathrm{HS}\;\mathrm{MRI}\sim \mathrm{PRS}+\mathrm{Sex}+\mathrm{Age}+\mathrm{Ag}e^{2}+\text{Education}\\ +\text{Diagnosis}+\mathrm{TIV}+\mathrm{PCs} \end{array}$$

Pearson correlations between each SNP included in the AD-PRS calculation and CA1 and subiculum volumes were used as an exploratory prioritization step. To account for multiple testing, FDR correction was applied across the 166 correlation tests and no associations remained significant after FDR correction. Nevertheless, SNPs meeting nominal significance criteria (Pearson’s r > 0.05 and *p* value < 0.05) were later employed as independent variants for the estimation of linear regression models adjusted by the same covariates as before:


$$\begin{array}{l} \mathrm{HS}\;\mathrm{MRI}\sim \mathrm{SNP}\;\text{dose}+\mathrm{Age}+\mathrm{Ag}e^{2}+\mathrm{Sex}+\text{Education}\\ +\text{Diagnosis}+\mathrm{TIV}+\mathrm{PCs} \end{array}$$

Results for both regression models were then combined across all seven cohorts with a common-effect meta-analysis (I^2^ < 75%) for CA1 and subiculum, using the inverse variance weighted approach implemented with the *meta* package in R (Schwarzer, 2007).

Sensitivity analysis, including leave-one-cohort-out analyses, analyses adjusted for *APOE* ɛ4 genotype, and analyses stratified by MCI and control status, were performed to account for heterogeneity across cohorts and to evaluate whether the observed associations are independent of established AD-related mechanisms.

### Co-regulatory network analysis

To identify co-regulatory networks of genes showing a possible association with HS-MRI in our exploratory analyses, we used GeneFriends (van Dam et al., 2015), a bioinformatics pipeline previously reported (Kleineidam et al., 2020), based on the Sequence Read Archive (SRA) (Leinonen et al., 2011) in all tissues. Next, WebGestalt (Liao et al., 2019) was used to identify potential enrichments in the clusters of AD-associated variants (Bellenguez et al., 2022) found using Pearson correlation with HS-MRI. We used over-representation enrichment analysis (ORA) (Khatri et al., 2012) with the Geneontology functional database of Biological Process (no Redundant) in humans using protein-coding regions of the genome as reference set. We used STRING v12.0 (Szklarczyk et al., 2021) in order to find known and predicted protein–protein interactions. False discovery rate (FDR) correction was applied using WebGestalt’s and STRING’s default Benjamini-Hochberg method.

### Association of the PRS with AD in the presence of concomitant brain pathologies

We previously used our autopsy-confirmed AD patients (*n* = 332), of whom 84% had at least one concomitant pathology, to perform an association analysis of an AD-PRS (based on 39 variants) with AD in the presence of concomitant brain pathologies adjusted for the first four genetic PCs as previously published (de Rojas et al., 2021). In this study, we replicated this association using the latest AD-PRS (based on 83 variants) (Bellenguez et al., 2022), and the same cohort previously used by de Rojas et al. (2021) of histopathological confirmed AD pathology alone and concomitant with different neurodegenerative dementias that affect hippocampal volume: cerebrovascular disease, LBD, microscopic vascular lesions, TDP-43 aggregates and HS-aging.

## Results

### AD-PRS and HS-MRI

In the meta-analysis presented here, we included data from seven different cohorts (ADNI-1, ADNI-2, BIOFACE, FACEHBI, EPAD, MOPEAD and VP), which consisted of 1,859 participants with MRI and array genotyping data. Their demographic composition was as follows: 54.8% were female, 35.8% had MCI and 64.2% were individuals without dementia (Table 1). The average age of the participants was 73.3 (±6.47) years. An AD-PRS was calculated for these participants, which ranged from −3.60 to 3.73 on a scaled min-max basis (Supplementary Figure 2). MCI showed a tendency toward a higher AD genetic load compared to controls (Supplementary Figure 2A). This trend may be due to significant differences in AD-PRS observed between *APOE* ɛ4 MCI carriers and controls (*p* value = 8.4 × 10^−05^; Supplementary Figures 2B,C).

**Figure 1:**
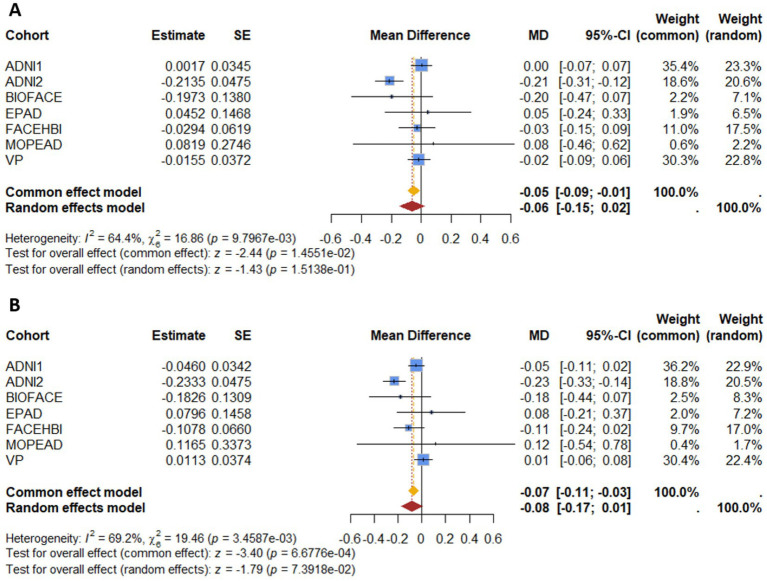
Meta-analysis for the association between AD-PRS and hippocampal subfield volumes. Forest plots for **(A)** CA1 and **(B)** subiculum.

This meta-analysis shows a significant association between the AD-PRS and both hippocampal subfield volumes referred to as HS-MRI after Bonferroni correction (*p* value < 0.025; Figure 1; Table 2): CA1 (*β* = −0.0501; SE = 0.0205; *p* value = 1.46 × 10^−02^) and subiculum (*β* = −0.0701; SE = 0.0206; *p* value = 6.68 × 10^−04^). These results suggest that participant without dementia (both MCI and cognitively healthy individuals) with higher genetic burden of AD have less volume in these hippocampal subfields.

**Table 2:** Meta-analysis results of the hippocampal subfield volumes association with AD-PRS (Bellenguez et al., 2022).

Hippocampal subfield	*β*	CI (95%)	*P* value
CA1	−0.05	−0.09, −0.01	**1.46 × 10** ^**−02**^
Subiculum	−0.07	−0.11, −0.03	**6.68 × 10** ^**−04**^

* Significant *p* values after Bonferroni correction in bold (*p* value < 0.025). † *β*, beta coefficient; CI, confidence interval.

Sensitivity analyses showed effect estimates consistent with the primary analysis and suggested that the association was mainly observed among participants with MCI. However, stratified analyses and adjustment for *APOE* ɛ4 did not meaningfully reduce heterogeneity across cohorts, while leave-one-out analyses indicated a contribution of the ADNI2 cohort to heterogeneity (Supplementary Table 1).

### AD SNPs and HS-by-proxy

**Figure 2:**
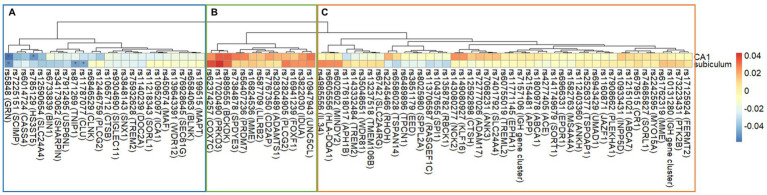
Correlations between CA1 and subiculum and AD SNPs included in the PRS in all study cohorts. **(A)** Framed in blue, cluster with a predominant negative Pearson’s correlation with CA1 and subiculum volumes represented in blue tones; **(B)** Framed in green, cluster with a predominant positive correlation with CA1 and subiculum volumes represented in orange and red tones; **(C)** Framed in orange, cluster with a mixed positive (red) and negative (blue) Pearson’s correlation with CA1 and subiculum volumes. All SNPs are represented by their risk allele for AD found by Bellenguez et al. (2022). Sig. level = 0.001***; 0.01**; 0.05*.

**Figure 3:**
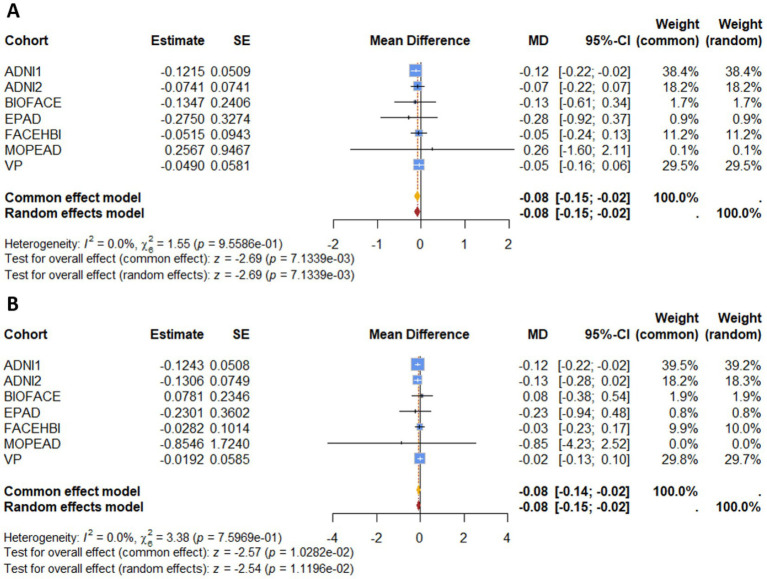
Meta-analysis for the association between *GRN* and hippocampal subfield volumes. Forest plots for **(A)** CA1 and **(B)** subiculum.

We tested for correlation with CA1 and subiculum and each of the 83 AD loci reported by Bellenguez et al. (2022) using Pearson’s correlation. This screening step was intended as an exploratory prioritization approach to reduce the dimensionality of AD loci and identify candidate variants for downstream analyses. No associations remained significant after FDR correction. However, variants in *GRN* (rs5848), *TNIP1* (rs871269), *HS3STS* (rs785129) and *CLU* (rs11787077) were found to have nominal correlations (*p* value < 0.05) with CA1 and/or subiculum volumes (Figure 2). Thus, we performed meta-analysis of the association of HS-MRI with each one of these four variants using the results obtained from each cohort (Table 3). The meta-analysis results show a significant association of *GRN* (rs5848) with CA1 [B = −0.085; SE = 0.0316, *p* value = 7.13 × 10^−03^] and subiculum [B = −0.082; SE = 0.0319, *p* value = 1.03 × 10^−02^] after Bonferroni correction (*p* value < 1.24 × 10^−02^; Figure 3).

**Table 3:** Meta-analysis results of the CA1 and subiculum association with the selected SNPs in the AD-PRS (Bellenguez et al., 2022).

Loci	SNP	*β*	CI (95%)	*P* value
CA1				
*GRN*	rs5848	−0.085	−0.15, −0.02	**7.13 × 10** ^**−03**^
*HS3ST5*	rs785129	−0.018	−0.08, −0.04	0.56
*CLU*	rs11787077	−0.042	−0.10, 0.02	0.17
*TNIP1*	rs871269	−0.051	−0.11, 0.01	0.11
Subiculum				
*GRN*	rs5848	−0.082	−0.15, −0.02	**1.03 × 10** ^**−02**^
*HS3ST5*	rs785129	−0.011	−0.07, 0.05	0.73
*CLU*	rs11787077	−0.060	−0.12, 0.00	5.35 × 10^−02^
*TNIP1*	rs871269	−0.071	−0.13, −0.01	2.62 × 10^−02^

* Significant *p* values after Bonferroni correction in bold (*p* value < 1.24 × 10^−02^). † SNP, single nucleotide polymorphism; *β*, beta coefficient; CI, confidence interval.

Additionally, Pearson’s correlation of HS-MRI with all 83 AD-PRS loci show unequal relations which divide the data in three differentiated clusters (Figure 2). Variants in cluster A (Figure 2) show an overall negative correlation with HS-MRI, meaning that they are generally correlated with smaller subiculum and CA1, thus potentially specific to tissue atrophy of hippocampal subregions associated with HS-aging. Notably, variants significantly correlated with HS-MRI (*GRN, TNIP1, HS3STS, CLU*) gather together in cluster A. In contrast, variants in cluster B and C (Figure 2) were not significantly associated with the phenotype and showed an overall positive correlation with CA1 and subiculum volumes, suggesting a tendency toward greater volumes of these hippocampal subregions related to HS-aging among carriers of these variants.

### Enrichment analysis of clusters of AD SNPs generated in association with HS-by-proxy

Using Webgestalt, we performed ORA on the 24 genes included in cluster A (Figure 2; blue), which includes *CLU*, *GRN*, *TNIP1* or *HS3ST5*. This analysis revealed a significant enrichment of inflammatory and immune-related pathways, driven by gene sets that include *GRN*. Specifically, enriched terms included neuroinflammatory response (GO:0150076; FDR = 6.22 × 10^−05^), leukocyte activation involved in inflammatory response (GO:0002269; FDR = 2.55 × 10^−04^), myeloid leukocyte activation (GO:0002274; FDR = 2.55 × 10^−04^), positive regulation of response to biotic stimulus (GO:0002833; FDR = 2.33 × 10^−03^), regulation of innate immune response (GO:0045088; FDR = 2.82 × 10^−03^), and positive regulation of defense response (GO:0031349; FDR = 4.40 × 10^−03^).

Cluster B gathers 13 variants located in 13 different genes. We found a tendency of these genes toward enrichment in immune and signaling or stress response pathways. However, none of these terms reached statistical significance. These included, stress-activated protein kinase signaling cascade (GO:0031098; FDR = 0.86), interleukin-6 production (GO:0032635; FDR = 0.86), immune response-regulating signaling pathway (GO:0002764; FDR = 0.86), myeloid leukocyte activation (GO:0002274; FDR = 0.86).

Cluster C comprises 45 variants mapped to 44 different genes. ORA revealed significant enrichment in pathways related to amyloid-*β* and protein processing, including amyloid-beta metabolic process (GO:0050435; FDR = 1.23 × 10^−04^), amyloid precursor protein metabolic process (GO:0042982; FDR = 3.61 × 10^−03^), membrane protein proteolysis (GO:0033619; FDR = 3.03 × 10^−03^), peptide catabolic process (GO:0043171; FDR = 3.24 × 10^−03^), peptidyl-tyrosine modification (GO:0018212; FDR = 3.61 × 10^−03^). Enrichment was also observed for immune-related processes, including leukocyte mediated immunity (GO:0002443; FDR = 3.61 × 10^−03^), mononuclear cell differentiation (GO:1903131; FDR = 3.03 × 10^−03^), regulation of hemopoiesis (GO:1903706; FDR = 4.02 × 10^−03^), as well as for pathways related to cell development and response to stress, such as positive regulation of cell development (GO:0010720; FDR = 3.03 × 10^−03^) and response to fluid shear stress (GO:0034405; FDR = 5.13 × 10^−03^).

### Network of genes associated with GRN

We used STRING to see whether *CLU*, *GRN*, *TNIP1* and *HS3ST5*, the four genes nominally correlated with HS-MRI (*p* value < 0.05), belong to the same biological pathway. We saw that only *CLU* and *GRN* are significantly co-expressed in a neuroinflammatory response pathway: microglial cell activation (GO:0001774, FDR = 0.04).

To increase our statistical power to perform ORA, we extracted the genes co-expressed with *GRN* using Genefriends. Significantly enriched pathways involving genes co-expressed with *GRN* were predominantly associated with immune function: phagocytosis (GO:0006909; FDR = 6.75 × 10^−33^), immune response-regulating signaling pathway (GO:0002764; FDR = 1.71 × 10^−20^), cell activation involved in immune response (GO:0002263; FDR = 2.56 × 10^−17^), regulation of lymphocyte activation (GO:0051249; FDR = 8.38 × 10^−17^), regulation of immune effector process (GO:0002697; FDR = 5.75 × 10^−16^), regulation of innate immune response (GO:0045088; FDR = 7.17 × 10^−16^), positive regulation of response to biotic stimulus (GO:0002833; FDR = 4.03 × 10^−15^), myeloid leukocyte activation (GO:0002274; FDR = 1.74 × 10^−14^), regulation of endocytosis (GO:0030100; FDR = 4.13 × 10^−14^), and leukocyte mediated immunity (GO:0002443; FDR = 4.32 × 10^−14^).

### Association of the PRS with HS-aging

**Figure 4:**
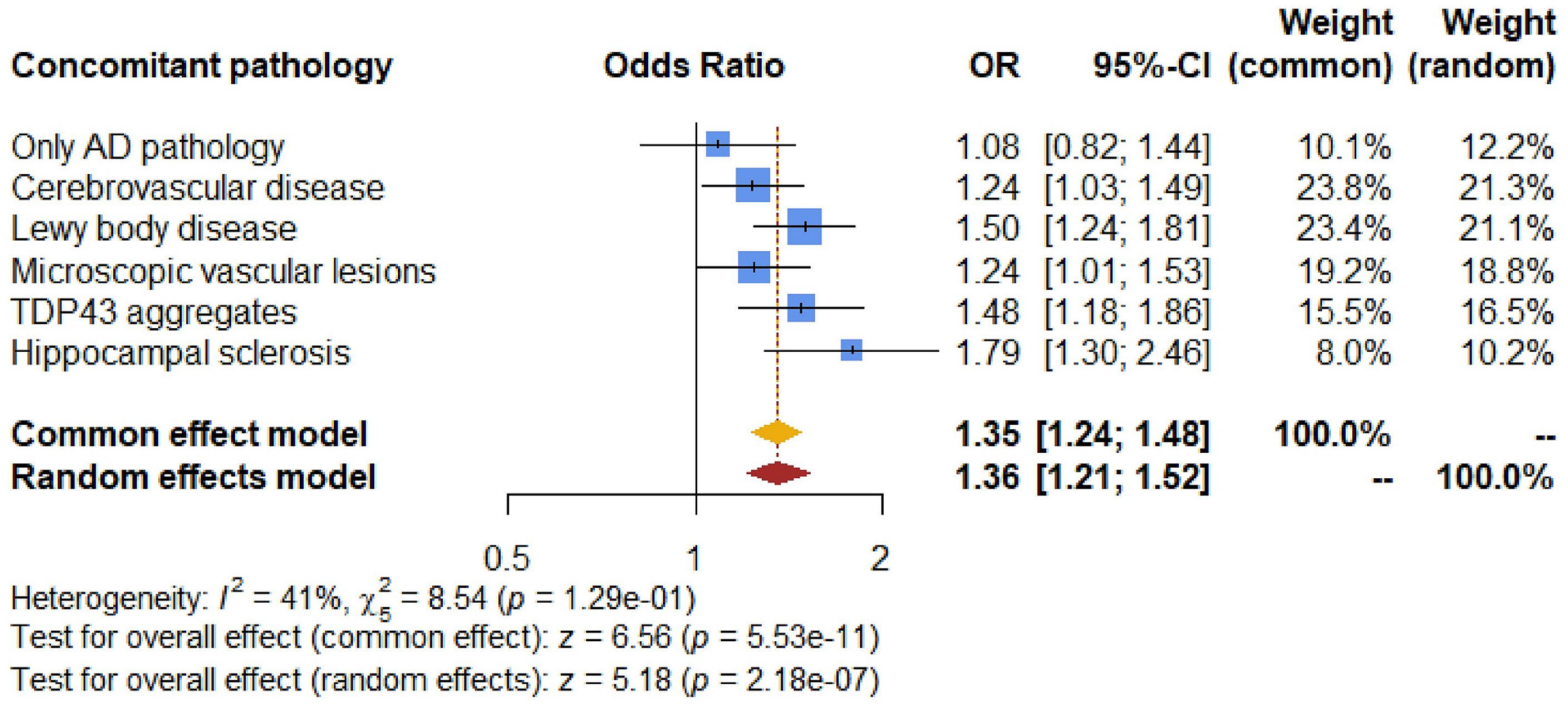
Meta-analysis for the association between AD-PRS and AD in the presence of concomitant brain pathologies. AD-PRS based on Bellenguez et al., 2022. Forest plots including data from EADB/ACE/BTN-Clinic-FRCB-IDIBAPS datasets from de Rojas et al. (2021). Data presented as OR per 1-SD increase in PRS (95% CI).

We replicated the association of AD-PRS with AD in the presence of concomitant neurodegenerative dementias. We previously reported this association using an AD-PRS calculated with 39 genomic variants (de Rojas et al., 2021). In this study, we base the AD-PRS on the latest 83 variants associated with AD (Bellenguez et al., 2022) and significantly associate it with AD in the presence of concomitant neurodegenerative dementias in a meta-analysis [I^2^ = 41%; OR (95% CI) = 1.35 (1.24–1.48), *p* value = 5.53 × 10^−11^; Figure 4].

Association analysis of this AD-PRS with each of the concomitant brain pathologies individually, including AD alone, was significant for LBD [OR (95% CI) = 1.50 (1.24–1.81), *p* value = 2.17 × 10^−05^], TDP43 aggregates [OR (95% CI) = 1.48 (1.18–1.86), *p* value = 7.76 × 10^−04^], and HS-aging [OR (95% CI) = 1.79 (1.30–2.46), *p* value = 3.84 × 10^−04^] after Bonferroni correction (*p* value < 0.0083; Table 4). Notably, AD-PRS was not significantly associated with AD pathology alone and the strongest effect was observed in HS-aging (Table 4).

**Table 4:** Association of AD-PRS (Bellenguez et al., 2022) and AD in the presence of concomitant brain pathologies.

Concomitant pathology	N	OR	CI (95%)	*P* value
Only AD pathology	54	1.08	0.82, 1.44	5.78 × 10^−01^
Cerebrovascular disease	145	1.24	1.03, 1.49	2.50 × 10^−02^
Lewy body disease	140	1.50	1.24, 1.81	**2.17 × 10** ^**−05**^
Microscopic vascular lesions	117	1.24	1.01, 1.53	3.96 × 10^−02^
TDP43 aggregates	87	1.48	1.18, 1.86	**7.76 × 10** ^**−04**^
Hippocampal sclerosis	43	1.79	1.30–2.46	**3.84 × 10** ^**−04**^

* Significant *p* values after Bonferroni correction in bold (*P* value < 8.33 × 10^−03^). † OR, odds ratio; CI, confidence interval; *N*, samples size.

## Discussion

Approximately 75% of older adults examined *post mortem* exhibit multiple co-occurring brain pathologies, commonly referred to as mixed neuropathologies (Brenowitz et al., 2016; Alzheimer’s Disease Research Center, 2022). This heterogeneity over time and across pathologies challenges genetic studies of AD. The large sample sizes required in GWAS often increase the inclusion of individuals with co-pathologies or uncertain diagnoses, and the resulting risk loci inevitably capture a mixture of genetic signals rather than pathology-specific effects. This mixing of signals, critically limits our ability to translate genetic findings into targeted therapeutic strategies and masks the specific biological mechanisms driving each pathology. The use of neuroimaging endophenotypes such as CA1 and subiculum volumes, which reflect early hippocampal changes compatible with HS-aging related processes (Woodworth et al., 2021; Ortega-Cruz et al., 2023), offers a more specific strategy to disentangle these mixed genetic signals and link individual risk loci to their underlying pathological mechanisms.

In line with previous research associating higher values of AD-PRS with lower hippocampal subfield volumes prior to dementia onset (Harrison et al., 2016; Foo et al., 2021; Kannappan et al., 2022), we demonstrate that higher AD-PRS values are associated with reduced CA1 and subiculum volumes in individuals without dementia. Moreover, when decomposing the AD-PRS into its constituent variants, we observe an uneven distribution of their association with CA1 and subiculum volumes. Particularly, our exploratory analyses suggest a possible association between variant rs5848 in *GRN* and CA1 and subiculum atrophy. This observation may point to a potential role of *GRN* in differential hippocampal subfield vulnerabilities prior to dementia onset. Consistent with previous findings (Jansen et al., 2022), this variant in *GRN* clusters with AD-PRS variants enriched for inflammatory and immune-related pathways, including *TNIP1* (rs871269), which shows significant Pearson’s correlation with CA1 and subiculum atrophy. Since both age and neurodegeneration are associated with inflammatory processes, we speculate that inflammatory responses in the hippocampus may influence autophagy events driven by these loci (Hammond et al., 2019; Zhang et al., 2023). However, given the exploratory nature of this finding, it should be interpreted with caution and requires independent replication.

This interpretation is further supported by our replication of the stronger association between AD-PRS and AD in the presence of concomitant neuropathologies compared with AD alone. We previously reported this association using an AD-PRS based on 39 variants in a Spanish histopathological cohort (de Rojas et al., 2021). Here, using the updated 83-variant AD-PRS (Bellenguez et al., 2022), our results confirm and extend this trend. AD-PRS shows its strongest association with histopathological diagnosed HS-aging. We speculate that part of the AD genetic signal reflects susceptibility to mixed pathology, specially HS-aging, rather than specifically to AD pathology. This strongest effect might be driven by variants involved in the immune-brain axis, such as rs5848 in *GRN* and rs871269 in *TNIP1*, which we also found to present a possible association here with a previously reported HS-aging endophenotype (Woodworth et al., 2021; Ortega-Cruz et al., 2023). However, alternative explanations should be considered. Mixed pathology may represent a more severe or advanced disease state that amplifies genetic associations regardless of their pathological specificity, and the small HS-aging sample size (n = 43) limits the strength of conclusions that can be drawn from this subgroup analysis.

The biological interpretation of these findings is supported by the established role of the implicated genes, although caution is needed given the observational design of the study. *GRN* (Granulin Precursor) encodes for granulins, glycosylated peptides which modulate inflammation in neurons, axonal outgrowth and neuronal integrity. Variant rs5848 in *GRN* has been specifically associated with HS-aging across multiple independent studies (Dickson et al., 2010; Katsumata et al., 2017; Shade et al., 2024). Similarly, rs871269 in *TNIP1*, implicated in NF-κB-dependent anti-inflammatory signaling, has been formerly associated with HS-aging pathology in a large European ancestry cohort (Katsumata et al., 2022). Our variant association and pathway enrichment analyses, together with prior HS-aging literature, are consistent with a potential role for these variants in HS-aging related processes, although they do not provide direct evidence of causality.

The use of hippocampal subfield volumes measured with MRI as a proxy for HS-aging is a clear limitation of this study. While CA1 and subiculum atrophy has been previously associated with HS-aging (Woodworth et al., 2021; Ortega-Cruz et al., 2023), they are not specific and may also reflect other AD-related neurodegeneration, LATE, or vascular processes. Even though hippocampal atrophy before dementia onset has been suggested as a useful early biomarker for HS-aging (Li et al., 2021, 2023; Woodworth et al., 2021), the interpretation of our results should be validated in individuals with a confirmed diagnosis of HS-aging. Therefore, our findings should be interpreted as indicative of hippocampal vulnerability patterns consistent with HS-aging, rather than as direct evidence of HS-aging pathology. A longitudinal follow-up of the present study to identify patients developing HS-aging, or future validations in HS-aging autopsy cohorts, would help further asses the relevance of our findings. The exploratory nature of the SNP correlation-based selection strategy and the downstream association and pathway analyses are also relevant limitations of this study. Although we lack statistical power to report significant results, our findings align with previous evidence and should be considered hypothesis-generating rather than confirmatory. Moreover, our limitations include the heterogeneity of the MRI data due to varying acquisition parameters and Freesurfer versions across cohorts. Although no formal cross-site harmonization method (e.g., ComBat) was applied to the imaging data, we attempted to reduce the impact of MRI variability across cohorts by normalizing MRI measures, excluding outlier values, adjusting analyses for TIV, and estimating associations using a meta-analytic framework. Different genotyping arrays also introduced heterogeneity across cohorts, which was addressed by conducting cohort specific association analysis and subsequently meta-analyzing the results. While these strategies reduce cohort-specific biases and improve the comparability of results across datasets, residual sources of heterogeneity are expected to influence the results due to variation in sample size, age, education or MCI proportion. Our sensitivity analyses do not show reduced heterogeneity when adjusting for APOE ɛ4, suggesting that observed associations are independent of this established genetic risk factor for AD. However, data on amyloid *β* and tau levels, as well as vascular burden, were not available for inclusion in our analyses. Therefore, we could not fully assess the independence of our results from AD-related mechanisms. The role of these factors in the observed associations requires further investigation. Despite limitations, our findings are consistent across independent cohorts and analytical approaches. However, they should be interpreted as consistent with HS-aging related vulnerability, rather than direct evidence of HS-aging pathology. AD-PRS shows a strong association with HS-aging and with CA1 and subiculum atrophy, suggesting that early hippocampal subfield atrophy may capture genetic signals relevant to this pathology. Specifically, variant rs5848 in *GRN*, previously related to HS-aging, may contribute to immune related processes that affect hippocampal subfield atrophy associated with the pathology. The etiology of HS-aging remains mostly unknown, and the absence of reliable biological markers for its detection makes it a subject of ongoing research. In this context, disentangling the genetic risk factors across different neuro-pathologies remains a necessary area of investigation. Our findings contribute to this effort, and suggest promising new directions for future research. Overall, this study highlights the importance of precise phenotyping in genetic studies to generate disease-specific PRS that might be key for premature detection. Dissecting the molecular pathways, cell types, and brain regions associated with each AD locus is crucial to translate genetic observations into clinical benefits.

## Data Availability

The data analyzed in this study is subject to the following licenses/restrictions: access to the private datasets must be requested directly from the respective principal investigators and is subject to their approval and data-sharing policies. Data from the Alzheimer’s Disease Neuroimaging Initiative (ADNI) are publicly available upon request through the ADNI data access procedures. Requests to access these datasets should be directed to vfernandez@fundacioace.org and https://adni.loni.usc.edu/.

## Glossary

ACE: Ace Alzheimer Center Barcelona
AD-PRS: AD polygenic risk score
AD: Alzheimer’s disease
ADNI: Alzheimer’s Disease Neuroimaging Initiative
Aβ: Amyloid β
Body and head (BH)
CA: Cornu Ammonis
DEGESCO: Dementia Genetics Spanish Consortium
TE: Echo time
EADB: European Alzheimer’s and Dementia Biobank (EADB)
EPAD: European Prevention of Alzheimer’s Dementia
FDR: False Discovery Rate
FOV: Field of view
FACEHBI: Fundació ACE Healthy Brain Initiative
GO: Gene Ontology
GR@ACE: Genome Research at Ace Alzheimer center
GWAS: Genome-wide association studies
GWS: Genome-wide significant
HS-aging: Hippocampal sclerosis of aging
TI: Inversion time
LATE: Limbic-predominant age-related TDP-43 encephalopathy
MRI: Magnetic resonance imaging
MPRAGE: Magnetization-prepared rapid gradient-echo
MCI: Mild cognitive impairment
MAF: Minor allele frequency
MOPEAD: Models of Patient Engagement for Alzheimer’s Disease
OR: Odds ratio
ORA: Over-representation enrichment analysis
PRS: Polygenic risk scores
PC: Principal Component
QC: Quality Control
TR: Repetition time
SRA: Sequence Read Archive
CeGEN: Spanish National Center for Genotyping
3D: Three-dimensional
TIV: Total intracranial volumes
TOPMed: Trans-Omics for Precision Medicine
VP: Vallecas Project
